# Computational Resources for Bioscience Education

**DOI:** 10.1007/s12010-021-03601-0

**Published:** 2021-06-08

**Authors:** Rajiv K. Kar

**Affiliations:** Faculty II-Mathematics and Natural Sciences, Technische Universität Berlin, Sekr. PC 14, Strasse des 17. Juni 135, D-10623 Berlin, Germany

**Keywords:** Bioscience, Structural modeling, Visualizers, Classical simulations, Quantum mechanics

## Abstract

With the ongoing laboratory restrictions, it is often challenging for bioscience students to make satisfactory progress in their projects. A long-standing practice in multi-disciplinary research is to use computational and theoretical method to corroborate with experiment findings. In line with the lack of opportunity to access laboratory instruments, the pandemic situation is a win-win scenario for scholars to focus on computational methods. This communication outline some of the standalone tools and webservers that bioscience students can successfully learn and adopt to obtain in-depth insights into biochemistry, biophysics, biotechnology, and bioengineering research work.

## Introduction

**Fig. 1:**
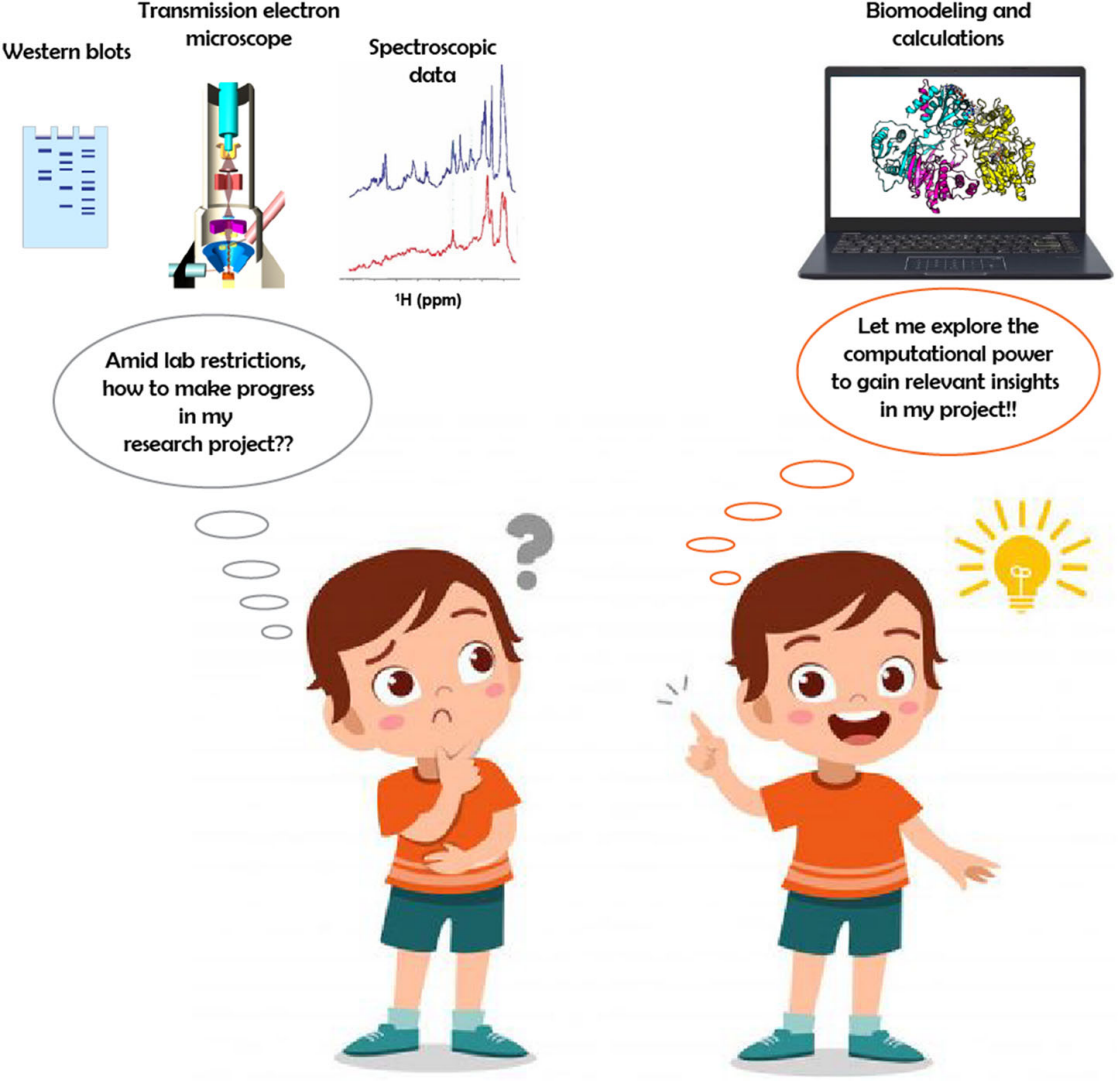
Illustration suggesting that exploring computational powers could be valuable for scholars to make progress in bioscience projects

The worldwide impact of COVID-19 has raised several challenges for research scholars [1]. The common problems include restricted access to lab workspace, delayed transportation of materials, and reduced technical assistance for experimental troubleshoot [2]. Though the online platforms have rescued to a certain level with one-on-one discussion, educational sessions, and virtual conferences, the problem still persists among the graduate students and forming the cloud of worries—“how to make progress in the research project?” (Fig. 1). While the laboratory experiments are not at full speed, it is still possible to use computational power. This communication aims to highlight a few useful computational tools that can help in making research progress, amid restricted laboratory access.

### Structural Modeling

Most of the bioscience problems deal with biological macromolecules such as proteins, nucleic acids, and small molecules. Though the structural coordinates are available from the protein data bank (PDB) for some structures, there exists a large sequence-structure gap for many biomacromolecules [3]. Homology modeling tools are a valuable starting point to analyze the putative three-dimensional conformation, interacting residues, and active site arrangement in these structures. The available standalone tools include MODELLER [4], while webservers are Phyre2 [5], ROBETTA [6], and SWISS MODEL [7]. The structural validation of such models for the interface analysis, surface assemblies, and Ramachandran plot can be determined using PDBePISA server [8]. The solvent accessibilities can also be analyzed using Naccess standalone program [9]. The contact map details can be analyzed using ConPlot [10] and DISTEVAL [11]. Relevant details of normal mode for predicting collective protein domain motions is achievable using iMODS [12], ElNémo [13], and WEBnm@ [14]. To determine the protein stability, especially for projects with protein mutants, one can use servers like SDM [15], MAESTROweb [16], PoPMuSic [17], DUET [18], and pPerturb [19]. These algorithms are accountable for predicting thermodynamics (free energy) properties based on input structural coordinates in a PDB format.

### Bio-macromolecular Complex

The macromolecular interactions are an important area that helps in better understanding protein functioning, mechanism, and drug designing. The widely used server ClusPro [20] is one of such tools that predict protein-interface based on the energetic evaluation. Hex program offers interactive fast Fourier transform-based docking [21]. Several useful options are also available on HADDOCK [22] webserver that can help in blind or experimental constraint-guided protein docking. While protein-protein docking is more complicated due to the presence of several degrees of freedom, the algorithm for small molecule docking is relatively simpler. The widely used AutoDock [23] is suitable for such exercise, with AutoDock Vina [24] offering high-throughput screening. Notably, these tools must be benchmarked for the system under consideration, such as by docking a known complex and comparing the root-mean-squared deviation (docked vs original complex). Additionally, the docking outcomes can be benchmarked against a reliable set of decoy molecular sets, available from DUD-E [25] and DEKOIS 2.0 [26] database. Schematic diagrams for protein-ligand contacts can be plotted using Ligplot [27]. More advanced tools such as for pKa prediction include Protein-Sol server [28], DelPhiPKa [29], and PDB2PQR [30]. However, these tools come with limitation to process non-standard residues such as small organic and drug-like molecules.

### Visualizers

**Fig. 2:**
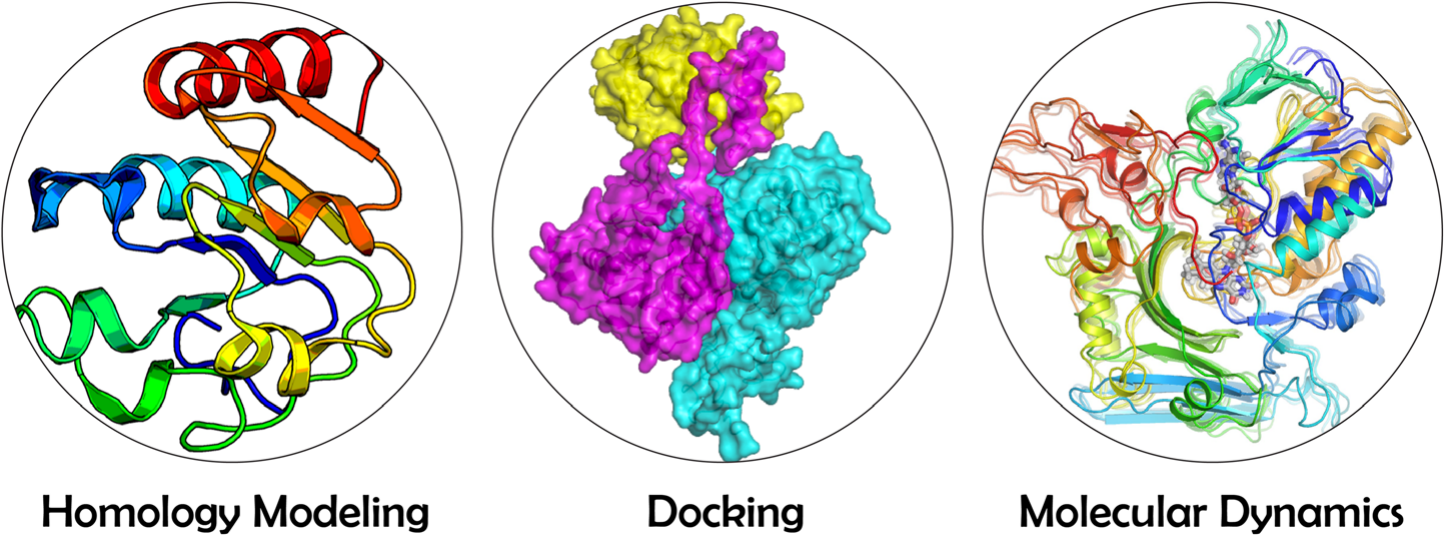
Representation of static structures from homology modeling and protein-protein docking, and dynamic structures from molecular dynamics simulation

The visualization tools add fun while depicting the spatial arrangement of amino acids and nucleobases, secondary structures, and water molecules in a three-dimensional network. Nevertheless, they can render publication-quality images (Fig. 2). One can also perform structural modeling with visualizers like PyMol [31], VMD [32], and Chimera [33].

### Classical Simulations

The collaboration between classical simulation with biophysics is more common these days. Some of the relevant free tools to perform molecular simulation include GROMACS [34] and Desmond [35]. However, they require advanced hardware to compute physics-based time-dependent motions of biomolecules. A Perl-based toolset for structure preparation and analysis is available with MMTSB [36]. While CHARMM-GUI server [37] provides numerous options to prepare solvated structures, membrane bilayer, and coarse-grain systems, one can also use this server to prepare input complement to other simulation engines. Equilibrated trajectories for a short time-scale are possible to be calculated using MDWeb server [38] and ChemCompute server [39]. Furthermore, scholars having a basic knowledge of python can make use of MDAnalysis [40] to extract information. Both VMD and Chimera offer capabilities to visualize, and varieties of plugins to analyze the simulation trajectory.

### Quantum Chemical Calculations

Calculations based on quantum mechanics (QM) are more accurate and reliable compared to the classical simulations; however, they come with their own set of limitations. Accurate modeling of protein-ligand interaction and elucidation of spectroscopic properties are some valuable insights that might be crucial to investigate biochemical and biophysical problems. Notably, rigorous quantum mechanics methods are mandated to achieve their transferability and robustness in connection to experimental corroboration. Orca [41] and GAMESS [42] are open-source quantum chemistry package that offer a wide range of capabilities, including geometry optimization, calculation of UV/Vis excitation energy, CD spectra, and vibrational frequencies. Avogadro [43] is user-friendly freeware that not only enables chemical editing but also to visualize frontier molecular orbitals and vibrational modes. Psi4 open-source package [44] is useful in analyzing the wavefunctions and Hartree-Fock energy decomposition. While these programs are accountable for high-computing time, one can run calculations with a smaller basis set on desktop and laptop with Windows or Linux operating system. Thankfully, it is also possible to perform QM calculations with a high basis set using the webserver ChemCompute [39], which offers computing time on cluster nodes for registered users with an academic electronic address.

## Conclusion

In summary, this communication highlights various (not limited to) computational tools relevant to obtain atomistic-scale analysis of biomacromolecules. While having restricted access to laboratory space and equipment, learning computational methods could mark a substantial advancement. Most of the standalone tool and web servers listed are freely accessible, with academic registration. Nevertheless, before using these computational tools and corroborating with the experimental measurements, it is essential to understand their theoretical principles.
